# The risk of urinary tract infection in vegetarians and non-vegetarians: a prospective study

**DOI:** 10.1038/s41598-020-58006-6

**Published:** 2020-01-30

**Authors:** Yen-Chang Chen, Chia-Chen Chang, Tina H. T. Chiu, Ming-Nan Lin, Chin-Lon Lin

**Affiliations:** 1 Department of Anatomical Pathology, Hualien Tzu Chi Hospital, Buddhist Tzu Chi Medical Foundation, Hualien, Taiwan; 2 Department of Medical Research, Hualien Tzu Chi Hospital, Buddhist Tzu Chi Medical Foundation, Hualien, Taiwan; 3 Department of Nutritional Science, Fu-Jen Catholic University, Taipei, Taiwan; 4 Department of Family Medicine, Dalin Tzu Chi Hospital, Buddhist Tzu Chi Medical Foundation, Chiayi County, Taiwan; 5 Department of Family Medicine, College of Medicine, Tzu Chi University, Hualien, Taiwan; 6 Department of Cardiology, Dalin Tzu Chi Hospital, Buddhist Tzu Chi Medical Foundation, Chiayi County, Taiwan; 7 Department of Internal Medicine, College of Medicine, Tzu Chi University, Hualien, Taiwan

**Keywords:** Urogenital diseases, Urethra

## Abstract

Urinary tract infection (UTI) is caused principally by ascending *Escherichia coli* infection via an intestine-stool-urethra route. Recent studies found that the strains of *E. coli* causing UTIs, called extra-intestinal pathogenic *E. coli* (ExPEC), were distinct from the intestinal pathogenic strains and normal commensal strains. Further analysis found the meat including poultry and pork is the major reservoir for ExPECs. Vegetarians avoid meat and should theoretically have less exposure to ExPEC. However, no study thus far has examined whether vegetarian diets reduce the risk of UTI. Our aim was to examine the association between vegetarian diet and UTI risk in a Taiwanese Buddhist population. We prospectively followed 9724 Buddhists free of UTI from 2005 to 2014. During the 10-year follow-up, 661 incident UTI cases were confirmed. Diet was assessed through a food frequency questionnaire. Cox regression was used to evaluate the prospective association between a vegetarian diet on risk of UTI while adjusting for age, sex, educational level, alcohol-drinking, smoking, hypertension, diabetes mellitus, hyperlipidemia, and disease conditions predisposing to UTIs. Overall, vegetarian diet was associated with 16% lower hazards (hazard ratio [HR]: 0.84, 95% confidence interval [CI]: 0.71–0.99). In subgroup analysis, the protective association between vegetarian diet and UTI is observed mainly in the female (HR: 0.82, 95% CI: 0.69–0.99), never smokers (HR: 0.80, 95% CI: 0.67–0.95), and for uncomplicated UTI (HR: 0.81, 95% CI: 0.68–0.98).

## Introduction

Urinary tract infection (UTI) is one of the most common microbial infections worldwide, with a global prevalence of 10 per 1,000 peoples. UTI is associated with considerable societal cost, significant morbidity, and even increasing antibiotic resistance, a current challenge for infection control^1,2^.

The route of infection is mainly through ascending infection from the distal urethra colonized by intestinal source microbes. The principal pathogens are Gram-negative bacteria, particularly the species of *Escherichia coli* accounting for 65~75% of all urinary tract infections, and the remaining are caused by Gram-positive bacteria or fungi^2^.

UTIs are categorized into two forms: uncomplicated UTI and complicated UTI, owing to different pathophysiology and management. Uncomplicated UTI is defined as individuals with UTI who are otherwise healthy and without any structural or neurological urinary tract abnormality that predisposes them to infection. In contrast, complicated UTI is associated with underlying factors that compromise urinary tract or host defense giving rise to predisposition to UTI, such as urinary obstruction caused by calculus or tumor, urinary retention by neurological disease, decreased urine output due to renal failure, renal transplantation, immune compromise or suppression, pregnancy, and indwelling catheter^2^.

Interestingly, recent studies found that the strains of *E. coli* causing UTIs, called extra-intestinal pathogenic *E. coli* (ExPEC), were distinct from the intestinal pathogenic strains and normal commensal strains, except some UTIs caused by the commensal *E. coli* in the complicated form (Table 1)^3,4^. Furthermore, analyses showed the isolates of *E. coli* from urine specimens of UTI patients were closely related to, if not indistinguishable from the pathogenic *E. coli* of poultry and pork^5–8^. This implicates that food reservoirs and foodborne transmission may play a role in the dissemination of ExPEC. As vegetarians avoid meat (including poultry and pork), we hypothesize that a vegetarian diet may be associated with a lower risk of UTI.

**Table 1:** The pathogenic potential of the three main different groups of *E. coli* strains^3,4^.

Clinical manifestation	Groups of *E. coli*		
	Commensal	ExPEC^a^	Intestinal pathogenic
Asymptomatic intestinal colonization	Major	Minor	None
Uncomplicated UTIs	Minor	Major	None
Complicated UTIs	50%	50%	None
Gastroenteritis	None	None	Major
Other infections other than UTI and gastroenteritis	Variable	Variable	None

^a^ExPEC: extra-intestinal pathogenic *E. coli*.

## Materials and Methods

### Study design and population

The Tzu Chi Vegetarian Study (TCVS) is a prospective cohort study that recruited 12062 Tzu Chi volunteers in 2005 throughout communities in Taiwan. Tzu Chi volunteers are devoted Buddhists who participate in a variety of charity works and disaster relief organized by the Buddhist Compassion Relief Tzu Chi Foundation. About one-third of this population became vegetarians in order to protect the environment and animals. Certified Tzu Chi volunteers are also required to quit smoking and alcohol-drinking.

The Buddhists Compassion Relief Tzu Chi Foundation has many liaison sites throughout Taiwan. In 2005, community leaders in all sites were asked to distribute research questionnaires to their local community volunteers, and send back these questionnaires upon completion. The questions included personal data, demographic information, educational level, medical history, and lifestyle habit including smoking, alcohol-drinking, physical activities, and diet. All participants had signed the informed consent on the first page of the questionnaire. This study was approved by the Institutional Review Board for ethics at Buddhist Dalin Tzu Chi Hospital.

### Assessment of disease status

We followed the TCVS participants from 2005 to 2014 by linking the baseline data to the National Health Insurance Research Database (NHIRD) and the death records at the National Health and Welfare Data Science Center (HWDC), Ministry of Health and Welfare.

In Taiwan, the National Health Insurance Program is a governmental universal health insurance program covering nearly 100% of the population. Individuals’ medical claim data including diagnosis from both inpatient and outpatient are available. In order to protect the privacy of individuals as required by local law, personal identification number is masked and all analyses must be performed within the HWDC. Only summarized research results (not individual data), could be released.

### Dietary assessment

The diet section in the questionnaire was adopted from the food frequency questionnaire (FFQ) used in Nutrition and Health Survey in Taiwan, and included 57 items on food and food groups, and a section on cooking methods and cooking oil. A similar questionnaire had been validated later and showed good reliability and validity among Tzu Chi volunteers^9^.

Participants were categorized as vegetarians if they (1) self-identified as vegetarians in a question asking vegetarian status, and (2) reported “no eating” in frequency questions for all individual meat and fish items in the FFQ. Those who self-identified as vegetarians but reported eating meat or fish in the FFQ were categorized as non-vegetarians.

### Exclusion criteria

**Figure 1:**
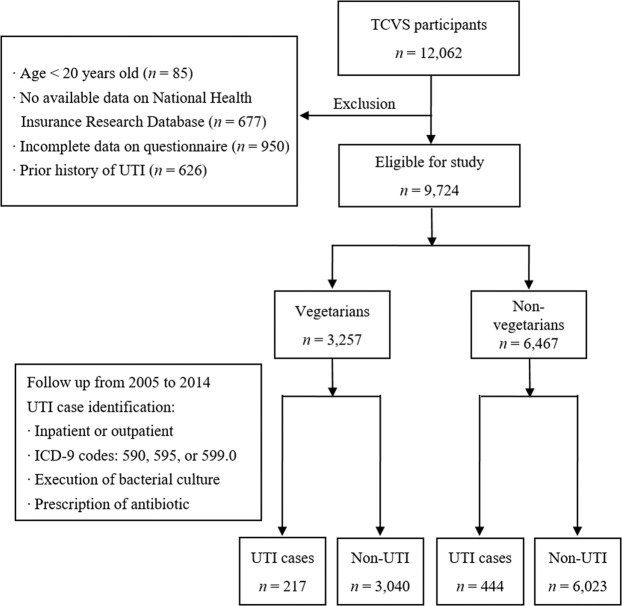
Chart showing the flow from TCVS to eligible study population and UTI case identification.

Individuals with age < 20 years old (*n* = 85), no available data on NHIRD (*n* = 677), incomplete data on the questionnaire (*n* = 950), and prior history of UTI (*n* = 626) were excluded from this study (Fig. 1).

### Case ascertainment

Incident cases of UTI were identified by the International Classification of Diseases, Ninth Revision (ICD-9) for UTIs and the codes for the execution of bacterial culture and prescription of antibiotics against UTI within the HWDSC database during follow-up.

The ICD-9 codes used include 590 for infections of the kidney, 595 for cystitis, and 599.0 for UTI, site not specified. The code for the execution of bacterial culture is 13007C. We identified UTI cases by the above ICD-9 diagnosis in either inpatient or outpatient, accompanied by the execution of bacterial culture and prescription of antibiotic against UTI^10,11^ (Fig. 1).

### Statistical analysis

Baseline characteristics of vegetarians and non-vegetarians were compared using Chi-square (χ^2^) test for categorical variables and independent sample *t* test for continuous variables.

Cox regression was used to analyze the association between different factors and risks of UTI, presented as hazard ratio (HR) with 95% confidence interval (CI). Model 1 adjusted for age and/or sex. Model 2 additionally adjusted for educational level and lifestyle factors (smoking and alcohol-drinking). Model 3 additionally adjusted for hypertension, diabetes mellitus, and hyperlipidemia. Model 4 additionally adjusted for disease conditions that predispose to complicated UTIs, including urinary tract obstruction caused by hyperplasia or tumor, urolithiasis (calculus), urine retention, renal failure, immune dysfunction, pregnancy, and urinary catheter indwelling. Kaplan-Meier curves of crude data were plotted to show the disease-free survival throughout follow-up period. Subgroup analyses by the above factors and test of interactions were performed. In addition to total UTI-combined, we also examined subtypes of UTI (complicated and uncomplicated) separately.

### Ethical approval

All procedures performed in studies involving human participants were in accordance with the ethical standards of the institutional and/or national research committee (Institutional Review Board [IRB] for ethics at Buddhist Dalin Tzu Chi Hospital with IRB approval document numbers: B09401003 and B10403020, grant numbers: TCMMPSP104-08-02, TCMMP105-13-05, TCMMP106-04-01) and with the 1964 Helsinki declaration and its later amendments or comparable ethical standards.

### Informed consent

Informed consent was obtained from all individual participants included in the study.

## Results

### Baseline characteristics

Table 2 shows the baseline characteristics of both vegetarian and non-vegetarian groups. The ratio of non-vegetarians to vegetarians was about 2:1. The vegetarian group was older, had a higher proportion of female, received less education, was less likely to have a history of smoking or alcohol-drinking, and had a significantly lower proportion of individuals with hypertension, diabetes, hyperlipidemia, and disease conditions that predispose to UTI.

**Table 2:** Baseline characteristics of vegetarian and non-vegetarian groups.

	Non-vegetarians	Vegetarians	P-value
	mean (std^a^)/*n* (%)	mean (std^a^)/*n* (%)	
Number of participants	6467 (66.5%)	3257 (33.5%)	
Age, years	50.1 (9.69)	51.2 (9.48)	<0.001
Sex			<0.001
Male	2677 (41.4%)	899 (27.6%)	
Female	3790 (58.6%)	2358 (72.4%)	
Educational level			<0.001
Elementary school or less	1277 (19.8%)	792 (24.3%)	
Secondary school	4503 (69.7%)	2086 (64.0%)	
College or higher	683 (10.5%)	383 (11.7%)	
Ever smoking	1126 (17.4%)	351 (10.8%)	<0.001
Ever alcohol-drinking	1064 (16.5%)	375 (11.5%)	<0.001
Hypertension	1154 (17.9%)	512 (15.7%)	0.008
Diabetes	466 (7.2%)	161 (4.9%)	<0.001
Hyperlipidemia	218 (3.4%)	79 (2.4%)	0.010
Disease conditions predisposing to UTI			
Hyperplasia/tumor of urinary tract	451 (7.0%)	160 (4.9%)	<0.001
Urolithiasis	743 (11.5%)	319 (9.8%)	0.011
Urine retention	132 (2.0%)	77 (2.4%)	0.306
Renal failure	135 (2.1%)	49 (1.5%)	0.049
Immune dysfunction	43 (0.7%)	23 (0.7%)	0.821

^a^Std: standard deviation.

### Risk factors associated with UTI

In the 10 years of follow-up, 661 individuals were diagnosed with UTIs. Table 3 shows the Cox regression analysis. Overall, UTI risk in vegetarians was significantly lower than non-vegetarians (HR = 0.84, 95% CI: 0.71–0.99, p = 0.038). Age, female sex, diabetes, urine retention, and renal failure were associated with significant increases in UTI risks. Hypertension, urinary obstruction (hyperplasia/tumor of the urinary tract), urolithiasis, and immune dysfunction were associated with non-significant higher UTI risks.

**Table 3:** UTI risks associated with vegetarian diet and other factors by Cox regression.

	Hazard ratios	95% CI^a^	P value
Vegetarians vs. non-vegetarians	0.84	0.71–0.99	0.038
Age	1.03	1.02–1.04	<0.001
Sex (Male vs. Female)	0.29	0.22–0.37	<0.001
Educational level			
Secondary school vs. elementary	0.92	0.77–1.11	0.399
College or higher vs. elementary	1.00	0.74–1.35	0.986
Ever smoking	1.11	0.80–1.56	0.534
Ever alcohol-drinking	1.16	0.85–1.58	0.357
Hypertension	1.19	0.97–1.45	0.094
Diabetes	1.57	1.21–2.05	0.001
Hyperlipidemia	1.16	0.78–1.71	0.463
Disease conditions predisposing to UTI			
Hyperplasia/tumor of urinary tract	1.47	0.99–2.19	0.055
Urolithiasis	1.22	0.97–1.53	0.094
Urine retention	1.86	1.28–2.72	0.001
Renal failure	1.97	1.36–2.88	<0.001
Immune dysfunction	1.87	0.97–3.63	0.063

^a^CI: confidence interval.

### Subgroup analysis

**Figure 2:**
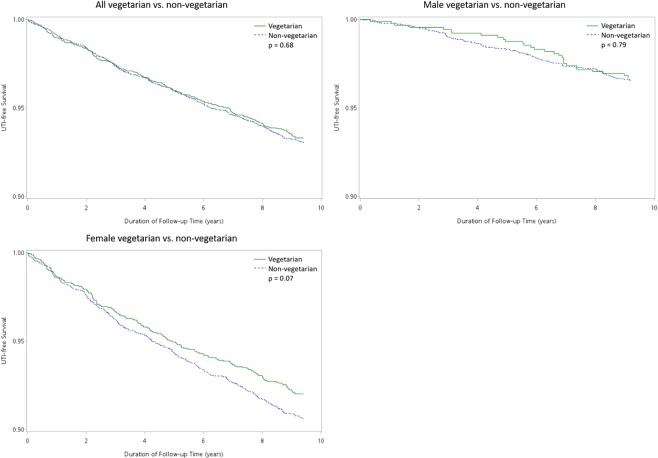
Kaplan-Meier curve of crude data over the ten-year period for vegetarians and non-vegetarians.

Subgroup analysis of the association between vegetarian diet and UTI by sex in various models were shown in Table 4. Vegetarian diet was significantly associated with a reduced risk of UTI mainly in female, particularly after adjustment for education and lifestyle (smoking and alcohol-drinking) in Model 2. Additional adjustments for hypertension, diabetes, hyperlipidemia, and conditions predisposing to UTI (as in Model 3 and 4) yielded similar results. Kaplan-Meier curves of UTI-free survival over the ten-year period for vegetarians and non-vegetarians (Fig. 2) highlight the protection of vegetarian diet in female, while no distinct difference was found in male.

**Table 4:** Hazard ratios (95% confidence interval) of UTI in different dietary and sex groups with different adjusted models by Cox regression.

	Vegetarians (*n* = 3257)			Non-vegetarians (*n* = 6467)		
	All	Male	Female	All	Male	Female
UTI cases	217	29	188	444	91	353
Person-years	29260	8204	21057	57882	24356	33526
Crude	0.97 (0.82–1.14)	0.95 (0.62–1.44)	0.85 (0.71–1.01)	1 (Reference)		
Model 1^a^	0.82 (0.70–0.97)	0.93 (0.61–1.41)	0.81 (0.68–0.97)			
Model 2^b^	0.82 (0.70–0.97)	0.94 (0.62–1.42)	0.81 (0.68–0.97)			
Model 3^c^	0.84 (0.71–0.98)	0.98 (0.64–1.48)	0.82 (0.69–0.98)			
Model 4^d^	0.84 (0.71–0.99)	0.98 (0.64–1.49)	0.82 (0.69–0.99)			

^a^Model 1: adjusted for age and/or sex.

^b^Model 2: additional adjustment for educational level and lifestyle (smoking and alcohol-drinking).

^c^Model 3: additional adjustment for hypertension, diabetes, and hyperlipidemia.

^d^Model 4: additional adjustment for disease conditions predisposing to UTI.

Additional subgroup analyses and the test for interactions are presented in Table 5. The test of interaction between dietary patterns and smoking was significant (P-interaction = 0.029), with the protection mainly among never smokers. No significant test of interaction was found between diet and other factors (sex, educational level, alcohol-drinking, hypertension, diabetes, hyperlipidemia, and disease conditions predisposing to UTI).

**Table 5:** Subgroup analysis of UTI risks in vegetarians versus non-vegetariansSubgroups.

	Number	Hazard ratio	95% CI^a^	P-interaction
Sex				0.486
Male	120	0.98	0.64–1.49	
Female	541	0.82	0.69–0.99	
Educational level				0.116
Elementary school or less	194	0.75	0.56–1.02	
Secondary school	406	0.84	0.68–1.04	
College or higher	61	1.27	0.76–2.13	
Smoking				0.029
Ever	62	1.52	0.87–2.65	
Never	599	0.80	0.67–0.95	
Alcohol-drinking				0.540
Ever	67	1.04	0.60–1.81	
Never	594	0.82	0.69–0.98	
Hypertension				0.177
With	133	1.09	0.76–1.57	
Without	528	0.79	0.66–0.95	
Diabetes				0.974
With	65	0.82	0.47–1.44	
Without	596	0.84	0.71–1.00	
Hyperlipidemia				0.955
With	27	0.75	0.31–1.81	
Without	634	0.84	0.71–1.00	
Disease conditions predisposing to UTI				
Hyperplasia/tumor of urinary tract				0.290
With	38	1.11	0.54–2.31	
Without	623	0.83	0.70–0.98	
Urolithiasis				0.755
With	86	0.77	0.48–1.24	
Without	575	0.85	0.71–1.01	
Urine retention				0.541
With	29	0.63	0.28–1.44	
Without	632	0.85	0.72–1.01	
Renal failure				0.606
With	29	0.79	0.32–1.95	
Without	632	0.84	0.71–1.00	
Immune dysfunction				0.532
With	9	0.80	0.08–8.30	
Without	652	0.85	0.72–1.00	

^a^CI = confidence interval.

The association between vegetarian diet and subtypes of UTI is shown in Table 6. Vegetarian diet was associated with significantly reduced risk of mainly the uncomplicated UTI but not complicated UTI.

**Table 6:** UTI risks of vegetarian diet in uncomplicated and complicated categories compared with non-vegetarian group by Cox regression.

	Uncomplicated UTI	Complicated UTI
All UTI cases	517	144
Hazard ratio (95% confidence interval)^a^	0.81 (0.68–0.98)	0.96 (0.67–1.37)
Male UTI cases	60	60
Hazard ratio (95% confidence interval)^a^	0.60 (0.30–1.18)	1.46 (0.84–2.52)
Female UTI cases	457	84
Hazard ratio (95% confidence interval)^a^	0.84 (0.69–1.02)	0.75 (0.48–1.19)

^a^Adjusted for age, sex, educational level and lifestyle (smoking and alcohol-drinking), hypertension, diabetes, and hyperlipidemia.

## Discussion

In this prospective cohort study, vegetarian diet (versus non-vegetarian diet) is associated with a lower risk of UTI particularly in female, non-smokers, and mainly for the uncomplicated-subtype of UTI. To the best of our knowledge, this is the first study that evaluated the prospective association between a vegetarian diet and UTI.

Previous research suggested that meat-related foodborne ExPECs may be the main cause of uncomplicated UTIs and a half of complicated UTIs (Table 1)^2–8^. Consistent with this finding, we found that the protective association between vegetarian diet and UTI risk was mainly in uncomplicated rather than complicated UTI, and this association is independent of diseases and risk factors predisposing conditions to complicated UTI including hypertension^12^, diabetes^13^, and hyperlipidemia^14^. This suggests that the effect of vegetarian diet is not mediated through these related diseases. The test of interaction showed that vegetarian diet (versus non-vegetarian diet) was associated with protection among the never smoking group, but not the ever smoking group (P-interaction = 0.029, Table 5). It is possible that smoking compensates the benefit of vegetarian diet by increasing blood pressures (which predisposing to renal failure)^12,15^, renal stone formation^16^, and urinary tract cancers^17^. All of which are also the disease conditions predisposing to complicated UTI^2–4^. However, the number of smokers in our study is small and the significant interaction could arise from chance that further confirmation from other studies is needed.

The principal pathogenic strains causing UTIs are ExPECs (accounting for 65~75% UTIs), a group of strains distinct from the intestinal pathogenic and normal commensal *E. coli*^3,4^. ExPECs express many characteristic virulent factors which are not present in other strains of non-ExPECs^2–4,18^. Many studies have illustrated that meat, including poultry and pork, as the major reservoirs for ExPECs^5–8,19^. Vincent *et al*. identified the *E. coli* strains by using O:H serotype and sequence type (ST) and discovered the O25:H4-ST131 and O114:H4-ST117 strains acquired from retail chicken were very similar to, if not indistinguishable from *E. coli* of UTI patients^5^. Jacobsen *et al*. performed the phylogenetic analysis by triplex polymerase chain reaction using three DNA markers showed the UTI-associated B2 and D phylogroups are found among isolates from broiler chicken meat, broiler chickens, pork, and pigs. Further, antimicrobial resistance phenotypes of *E. coli* from meat were very similar to isolates from UTI patients^7^. In addition, sequencing analysis of ExPECs showed UTI-causing ST10, ST69, ST95, ST117, and ST131 are associated with poultry and retail poultry meat^8,19^. Vegetarians do not consume meat and therefore avoid uptake of animal-food-associated ExPECs.

Recent studies discovered the gut and fecal microbiotas were different between vegetarians and non-vegetarians^20–23^. The high fiber contents in vegetarian diet may modulate the intestinal microbiota in humans^20,21^. Fiber is metabolized by intestinal anaerobic microflora to produce short-chain fatty acids, which decrease intestinal pH^20,21^. Vegans or vegetarians were shown to have lower total counts of *E. coli* and *Enterobacteriaceae spp*. and lower stool pH than non-vegetarians, and the lower stool pH could prevent the growth of *E. coli* and *Enterobacteriaceae*^22^. The shifts in the gut and fecal microbiotas in vegetarians may potentially contribute to the protection from UTI.

Vegetarians consume predominantly plant foods and with more frequent consumption of vegetables, fruits, and nuts than non-vegetarians in most cohorts^24,25^, as in our cohort study, published previously^26^. Briefly, vegetarians in this present study had previously been shown to have a distinct overall dietary pattern, with a more frequent consumption of a variety of vegetables, fruits, nuts, soy products and beans, while avoiding all types of meat, fish, and seafood, compared with non-vegetarians^26^. Plant foods contain phytochemicals (major classes including terpenoids, phenolics, alkaloids) which have been demonstrated to have antibacterial activities^27–40^, in addition to anti-carcinogenic, anti-mutagenic, anti-inflammatory, and anti-oxidative factors^27^. Among the phytochemicals, phenolics compounds are related to the antibacterial activities^27–40^, and are abundant in vegetables, whole grains, fruits, and nuts^28,29^, and some of which had been demonstrated with antimicrobial effect^27,30–40^. Cranberry (*Vaccinium macrocarpon*), belong to the *Ericaceae* family, is one of the most well-known fruit against UTI as evidenced in many trials^30–36^. Besides cranberry, fresh juice (berry or fruit) have been was associated with a decreased risk of recurrence of UTI (odds ratio: 0.66, 95% CI: 0.48–0.92, per 2 dL juice)^33^. Proanthocyanidins (PAC), a member of tannins belong to phenolics found in cranberry and other fruits may defend against microbes^30^. PAC inhibits the adhesion of P fimbriae *in vitro* via conformational changes and decrease in adhesion forces^30,34,36^, and this *in vitro* anti-adherence effect is dose-dependent^30^. Moreover, fructose found in fruits may also inhibit the adherence of type 1 fimbriae^30^. These fimbriae-mediated adhesions are the key step for infection^2^. In addition to ExPEC, *in vitro* studies have shown that cranberry may inhibit adherence of other common UTI pathogens including *Klebsiella pneumoniae*, *Staphylococcus aureus*, *Proteus spp*., *Pseudomonas aeruginosa*, and *Enterococcus faecalis*^30,36^. Clinical research suggests the dose of cranberry as follows^30^: (1) cranberry juice cocktail 240–300 ml daily (preventing 50% of recurrences of UTIs and reducing bacteriuria), (2) dried, concentrated juice extract 600 to 1,200 mg per day divided into two or three daily doses, or (3) total 36 mg of PAC in twice-daily manner. In addition to cranberry, other berries and fruits, such as elderberry (*Sambucus nigra*), blueberry (*Vaccinium corymbosum*), strawberries (*Fragaria* *ananassa*), blackberries (*Rubus spp*.), red raspberry (*Rubus idaeus*), blackcurrant (*Ribes nigrum*), and redcurrants (*Ribes rubru*m), also contain phenolics and may have similar properties against p-fimbriated *E. coli*^37^. Another plant with studies showing an effect against UTI is roselle (*Hibiscus sabdariffa*)^38–40^. Roselle, commonly consumed in the form of tea or jam in Taiwan, belongs to the *Malvaceae* family and is rich in phytochemical phenolics especially anthocyanins^38^. Alshami *et al*. had demonstrated the antimicrobial activity of roselle extract against uropathogenic *E. coli*, *K. pneumoniae*, and *Candida albicans* isolated from recurrent urinary tract infection, through inhibiting the biofilm forming capacity^39,40^. Moreover, commonly consumed nuts including almond, Brazil nut, cashew, chestnut, hazelnut, heartnut, macadamia, peanut, pecan, pine nut, pistachio, and walnut also contain a variety of phytochemicals including PAC, although there is no study demonstrating the antibacterial effect so far^41^. Studies about medicinal plants had shown many of them with broad spectrum antimicrobial activity against uropathogenic *E. coli* and other UTI pathogens^42,43^. However, the component contributing to the effect remains to be investigated. In sum, vegetarian diets comprise abundant phytochemicals, which may contain anti-microbial properties and protect against UTI.

ExPECs isolated from animal food products and UTI patients are very similar in antibiotic resistant patterns and virulence factor profiles^5,6,8,44,45^. Antibiotics use in animal agriculture may contribute to the increasing antibiotic resistance in humans^44^. The extended spectrum β-lactamase (ESBL)-producing and fluoroquinolone-resistant ExPECs, most of which are globally disseminated *E. coli* strain O25:H4-ST131 (accounting for 78%), are a major problem with resistance to penicillins, cephalosporins, and fluoroquinolones^2,8,46^. Moreover, the ESBLs encoded on plasmids typically carry other antibiotic resistance genes against aminoglycosides, sulfonamides, and quinolones, leading to multidrug resistant^2^. The high recurrence rates and increasing multidrug resistance in ExPECs^2^ make antibiotic treatment of UTI challenging. Therefore, alternative non-antibiotic management for UTI is important. High phytochemical content in a healthy vegetarian diet may provide an alternative prophylaxis from and bactericidal effect against UTI^27,30–40^, and a study had demonstrated the PAC from cranberry also exhibited the anti-adherence property in the multidrug resistant strains of uropathogenic P-fimbriated *E. coli*^47^.

Several limitations must be noted in our study. (1) Our case definition is based on ICD-9 code rather than the gold standard, which should be clinical symptoms and laboratory examination including pyuria and urine culture with a more than 100,000 colony forming units per milliliter^48,49^. (2) Residual confounding may still remain as several known confounders, including water intake^50^, sexual intercourse^33,51^, intake of honeydew melon (which may also contain ExPEC^5^, though evidence is insufficient), were not measured. (3) Although ExPEC is found mainly from meat sources, one study reported the detection of ExPEC isolates from a honeydew melon sample of restaurant/ready-to-eat foods in North America^5^. To the best of our knowledge, *E. Coli* or ExPEC had never been reported in plant based foods in Taiwan. The sporadic case of ExPEC in non-meat product may be due to contamination and will require further research. However, if plant-based foods were a major source of ExPEC contributing to UTI, the protective association between vegetarian diet and UTI would have been attenuated, strengthening the null association. (4) Another limitation lies in our FFQ, which did not ask specifically about intake of honeydew melon, cranberries, or roselle. Thus, we were unable to conduct further analysis on the association between UTI and these plant foods. In addition, the FFQ was self-administered with some degree of missing values, that it is difficult to calculate the exact intake of specific phytochemicals known to contribute to UTI-protection.

In conclusion, vegetarian diet is protectively associated with UTI particularly in female and for uncomplicated UTI. Further study with identification of pathogens from urine culture is needed to clarify the relationship among UTI risk, pathogens, and vegetarian diet.

## Data Availability

The data for this study (including National Health Insurance Research Database of Taiwan) is located at the Health and Welfare Data Science Center, Ministry of Health and Welfare. Access to these data requires an application to the Health and Welfare Data Science Center, Ministry of Health and Welfare, as per local law and regulation.
